# Simultaneous Quantification of *Plasmodium* Antigens and Host Factor C-Reactive Protein in Asymptomatic Individuals with Confirmed Malaria by Use of a Novel Multiplex Immunoassay

**DOI:** 10.1128/JCM.00948-18

**Published:** 2019-01-02

**Authors:** Ihn Kyung Jang, Abby Tyler, Chris Lyman, Maria Kahn, Michael Kalnoky, John C. Rek, Emmanuel Arinaitwe, Harriet Adrama, Maxwell Murphy, Mallika Imwong, Clare L. Ling, Stephane Proux, Warat Haohankhunnatham, Melissa Rist, Annette M. Seilie, Amelia Hanron, Glenda Daza, Ming Chang, Smita Das, Rebecca Barney, Andrew Rashid, Jordi Landier, David S. Boyle, Sean C. Murphy, James S. McCarthy, François Nosten, Bryan Greenhouse, Gonzalo J. Domingo

**Affiliations:** a Diagnostics, PATH, Seattle, Washington, USA; b Quansys Biosciences, Logan, Utah, USA; c Infectious Diseases Research Collaboration, Kampala, Uganda; d Department of Medicine, University of California at San Francisco, San Francisco, California, USA; e Faculty of Tropical Medicine, Department of Molecular Tropical Medicine and Genetics, Mahidol University, Bangkok, Thailand; f Faculty of Tropical Medicine, Mahidol-Oxford Tropical Medicine Research Unit, Shoklo Malaria Research Unit, Mahidol University, Mae Sot, Thailand; g QIMR Berghofer Medical Research Institute, Brisbane, Australia; h Department of Laboratory Medicine, University of Washington, Seattle, Washington, USA; i Department of Microbiology, University of Washington, Seattle, Washington, USA; j Center for Emerging and Re-emerging Infectious Diseases, University of Washington, Seattle, Washington, USA; k Nuffield Department of Medicine, Centre for Tropical Medicine and Global Health, University of Oxford, Oxford, United Kingdom; Cepheid

**Keywords:** malaria, *P. falciparum*, *P. vivax*, diagnostics

## Abstract

Malaria rapid diagnostic tests (RDTs) primarily detect Plasmodium falciparum antigen histidine-rich protein 2 (HRP2) and the malaria-conserved antigen lactate dehydrogenase (LDH) for P. vivax and other malaria species. The performance of RDTs and their utility is dependent on circulating antigen concentration distributions in infected individuals in a population in which malaria is endemic and on the limit of detection of the RDT for the antigens. A multiplexed immunoassay for the quantification of HRP2, P. vivax LDH, and all-malaria LDH (pan LDH) was developed to accurately measure circulating antigen concentration and antigen distribution in a population with endemic malaria. The assay also measures C-reactive protein (CRP) levels as an indicator of inflammation. Validation was conducted with clinical specimens from 397 asymptomatic donors from Myanmar and Uganda, confirmed by PCR for infection, and from participants in induced blood-stage malaria challenge studies. The assay lower limits of detection for HRP2, pan LDH, P. vivax LDH, and CRP were 0.2 pg/ml, 9.3 pg/ml, 1.5 pg/ml, and 26.6 ng/ml, respectively. At thresholds for HRP2, pan LDH, and P. vivax LDH of 2.3 pg/ml, 47.8 pg/ml, and 75.1 pg/ml, respectively, and a specificity ≥98.5%, the sensitivities for ultrasensitive PCR-confirmed infections were 93.4%, 84.9%, and 48.9%, respectively. Plasmodium LDH (pLDH) concentration, in contrast to that of HRP2, correlated closely with parasite density. CRP levels were moderately higher in P. falciparum infections with confirmed antigenemia versus those in clinical specimens with no antigen. The 4-plex array is a sensitive tool for quantifying diagnostic antigens in malaria infections and supporting the evaluation of new ultrasensitive RDTs.

## Abstract (precis)

Malaria rapid diagnostic tests (RDTs) primarily detect Plasmodium falciparum antigen histidine-rich protein 2 (HRP2) and the malaria-conserved antigen lactate dehydrogenase (LDH) for P. vivax and other malaria species. The performance of RDTs and their utility is dependent on circulating antigen concentration distributions in infected individuals in a population in which malaria is endemic and on the limit of detection of the RDT for the antigens.

## INTRODUCTION

Low-density malaria parasite infections are highly prevalent in low-transmission areas, often presenting in individuals with no clear malaria symptoms but with high potential for transmission of parasites (1–3). The availability of tools to better characterize these low-density parasite infections is important to accelerate and measure progress of malaria elimination.

The most widely used malaria diagnostic methods, such as blood film microscopy and rapid diagnostic tests (RDTs), have insufficient sensitivity to detect low-density parasite infections (4). Molecular tests detecting parasite nucleic acids, such as PCR tests, are more sensitive, achieving limit(s) of detection (LOD) of 0.1 parasites/µl (5–10). However, parasite antigen detection is relatively quick and simple to conduct. Multiplex immunoassays offer a means to detect multiple targets from the same blood aliquot while requiring only a small volume of sample and materials, as opposed to a conventional enzyme-linked immunosorbent assays (ELISAs), which target a single antigen.

Current immunoassays for detection of malaria infection typically target histidine-rich protein 2 (HRP2) or Plasmodium lactate dehydrogenase (pLDH) (11, 12). pLDH is highly conserved across all human malaria species, and is therefore used as an indicator of infection for all human malaria species. pLDH also has Plasmodium species-specific epitopes, enabling discrimination of Plasmodium species (13). C-reactive protein (CRP), a human inflammation marker, has been investigated as a biomarker to differentiate, in combination with malaria diagnosis, viral from nonviral infection in febrile case management (14, 15).

Fluorescent bead-based assays have been developed to support creation of more sensitive assays for HRP2 (16). A sensitive chemiluminescence-based multiplexed assay format has been developed to simultaneously detect HRP2 and CRP in the context of a multiplexed micronutrient ELISA (17). A 2-plex array derived from this has been used as a reference for the evaluation of an ultrasensitive HRP2-based RDT (18, 19). The 2-plex array was recently expanded into a 4-plex array to include assays that quantify HRP2, pan LDH (all malaria), and P. vivax LDH and to monitor host inflammatory response via CRP.

This study describes the validation of the 4-plex array. This 4-plex assay was then used to investigate the antigen concentration distributions and CRP in asymptomatic malaria infections.

## MATERIALS AND METHODS

### Ethics.

All specimens used in this study have been described previously (18, 19). Whole-blood EDTA specimens were collected from consenting study participants or with the assent of children and consenting legal caregivers within the context of studies approved by the relevant institutional review boards (IRBs). The Uganda study was approved by the University of California at San Francisco (IRB approval number 11-05995), Makerere University (IRB approval number 2011-0167), and the London School of Hygiene and Tropical Medicine (IRB approval number 5943). The Myanmar study was approved by the University of Oxford Tropical Research Ethics Committee (reference numbers 1017–13 and 1015–13), Tak Community Advisory Board, and by relevant village committees. The induced blood-stage malaria (IBSM) challenge studies were approved by the QIMR Berghofer Medical Research Institute (IRB approval numbers 2080, 2092, 2098, and 2142), and clinical trial registrations (ClinicalTrials.gov registration numbers NCT02389348, NCT02431637, NCT02431650, and NCT02573857).

### Clinical studies and specimens.

In Nagongera (Tororo District, Uganda), 100 random households were selected and visited to recruit children from the ages of 6 months to 11 years, as well as their primary caregivers, into a surveillance cohort, as previously described (20). In a Karen village (TOT), Myanmar, study teams visited households and recruited both children and adults as part of an ongoing study to assess mass drug administration (MDA) for malaria elimination. IBSM challenge studies were performed at QIMR, Queensland, Australia. In the IBSM studies, healthy volunteers with no recent history of malaria were inoculated intravenously with approximately 1,800 to 2,800 3D7 P. falciparum-parasitized red blood cells on day 0. On day 7, participants were admitted to the study unit, treated with an antimalarial, and observed for at least 72 h. All specimens were processed and stored at −80°C prior to any molecular and antigen testing.

### Reagents.

Recombinant GST-HRP2 W2 protein was purchased from Microcoat Biotech (Starnberger See, Germany), and recombinant P. falciparum LDH and P. vivax LDH proteins were purchased from CTK Biotech (San Diego, CA), and MyBioSource (San Diego, CA). CRP was from HyTest (Turku, Finland). Monoclonal antibodies recognizing P. falciparum HRP2, P. falciparum LDH, and P. vivax LDH were obtained from commercial vendors, and the custom anti-HRP2 antibodies were produced under contract at Precision Antibody (Columbia, MD) (21). The details of the antibodies are listed in Table S1 in the supplemental material. Anti-CRP (C6) antibody was purchased from HyTest (Turku, Finland).

### Reference materials.

P. falciparum-parasitized red blood cells from culture-adapted strain ITG (ATCC) were used as HRP2 and pLDH reference material. P. falciparum parasites were cultured in type O+ human erythrocytes diluted at 4% hematocrit in RPMI 1640 medium supplemented with HEPES (Gibco), sodium bicarbonate (Sigma), hypoxanthine (Sigma), gentamicin (Sigma), and either 10% human serum (Interstate Blood Bank) or 0.5% AlbuMax II (Invitrogen), using a modified *in vitro* culture technique (22). Parasite cultures were synchronized with 5% d-sorbitol three times prior to harvesting them at ring stage (>99%), and the parasitemia of the culture was determined by microscopic analysis (23). Culture reference materials prepared at 200 parasites/μl in EDTA-anticoagulated whole blood (Interstate Blood Bank) were frozen for long-term storage at −80°C. HRP2 and pLDH within reference material were quantified using the Malaria Ag competitive ELISA (CELISA) kit (Cellabs, Sydney, Australia) and the Qualisa malaria kit (Tulip Group, India), respectively. ELISAs were performed according to manufacturer protocols, with the following modification: for HRP2 ELISA, the incubation steps with antigen and horseradish peroxide (HRP) conjugate were performed at 37°C instead of room temperature, as it was found that the higher temperature increased the assay sensitivity. The accuracy of this assay was confirmed with a positive control at the known concentration of HRP2. Recombinant GST-HRP2 W2 (Microcoat Biotech, Starnberger See, Germany), recombinant P. falciparum LDH (MyBioSource, San Diego, CA), and recombinant P. vivax LDH (MyBio Source, San Diego, CA) were used on each ELISA kit to make standard curves.

### Human malaria 4-plex array development.

PATH (Seattle, WA, USA) developed a partnership with Quansys Biosciences (Logan, UT, USA) to develop a customized human malaria 4-plex array designed to test blood specimens.

### Phase I: antibody screening.

Q-Plex technology is based on printing capture antibodies in 350- to 500-μm spots at the surface of polypropylene 96-well plate to capture target antigens (see http://www.quansysbio.com/assay-development/). Fourteen anti-HRP2, five anti-pan LDH, and three anti-P. vivax LDH antibodies from different vendors were evaluated in custom spotted micro wells to identify the best-performing antibody pairs for each target antigen, HRP2, pan LDH, or P. vivax LDH. The best-performing mouse monoclonal capture and detection antibodies recognizing Plasmodium antigens were selected from comprehensive checkerboard antibody pairing against recombinant proteins at 10 pg/ml for HRP2 and 1,000 pg/ml for pan LDH and P. vivax LDH. The signal-to-noise ratio was calculated with chemiluminescence signals from antigen wells and blank wells.

### Phase II: assay optimization.

Assay conditions, including capture antibody printing conditions, calibrator curves, calibrator or sample diluent formulations, detection conditions, assay protocol, and reagent storage conditions (including antigen lyophilization) were optimized to maximize sensitivity and specificity of the assay. Calibrator concentrations were confirmed using HRP2 and pLDH concentrations within reference material measured by two commercial ELISA kits.

### Phase III: performance verification.

The protocols and procedures from phase II were verified with a lot of product produced in this phase. Company verification parameters included precision, dilutional linearity, edge effect, drift effect, interference from rheumatoid factors, sensitivity, and specificity.

### Human malaria 4-plex array.

On the day of analysis, frozen blood samples were thawed and kept on ice until use. Calibrators and samples were prepared according to the manufacturer’s protocol. After addition of 50 µl of calibrators and samples, the plate was incubated at room temperature with shaking at 500 rpm for 2 h. Plates were then washed with proprietary wash buffer using an automated plate washer. A 50-µl aliquot of detection mix containing proprietary ingredients, including biotinylated antibodies and buffer, was added to each well, and the plate was incubated with shaking for another hour and then washed again. For detection, a 50-µl aliquot of horseradish peroxide (HRP)-conjugated streptavidin solution was added to each well and then incubated with shaking for 30 min. After a final wash, a 50-µl aliquot of chemiluminescent substrate solution was added to each well and the chemiluminescent intensity from the array spots in each well was immediately measured using the Q-View Imager Pro (Quansys Biosciences) at an exposure time of 300 s.

### Assay validation specimens.

Five specimens, including P. falciparum cultures and P. vivax-infected blood, were used to assess dilutional linearity. Accuracy and precision were determined using a set of specimens spiked with exogenous HRP2, P. vivax LDH, and CRP at three different concentrations within the assay dynamic range. Diagnostic sensitivity and specificity were determined with clinical specimens from Myanmar and Uganda, described previously (18). Based on the results of quantitative PCR (qPCR), three categories of specimens were selected for evaluation of the 4-plex array, as follows: specimens with P. vivax infection (*n* = 94); specimens with P. falciparum infection (*n* = 106); and negative specimens (*n* = 197). The 4-plex array was also performed on IBSM challenge study specimens (18). P. falciparum parasite density in these specimens was measured by qPCR targeting 18S rRNA, as described previously (24). All clinical specimens from the field and IBSM studies were independently randomized, and the assay user was blinded to the specimen origin.

### Theoretical limit of detection and quantification of the assay.

Calibrator was prepared in the specialized diluent containing additives that equalize or minimize any differences between blood and calibrator diluent to generate the standard curve of the ELISA. The theoretical lower limit of detection (LLD) is the lowest concentration of analyte that can be detected in a diluted liquid specimen with a specific degree of probability. LLD was calculated using the following formula: LLD = 2 × (standard deviation of negative-control pixel intensities before negative well subtraction) × lower limit of quantification (LLOQ)/(difference between pixel intensity of lowest standard and negative control). The LLOQ and upper limit of quantification (ULOQ) are defined as the lowest calibrator point and the highest calibrator point, respectively, for which the concentration can be back-calculated on the nonlinear regression curve with 80% to 120% accuracy and a coefficient of variation (CV) of less than 30%. The prozone effect was also evaluated as presence of false-negative or false-low results due to presence of excess amounts of antigens or antibodies in immune reactions (25).

### Dilutional linearity.

Dilutional linearity of the 4-plex array was assessed by diluting five high-concentration blood specimens, including three P. falciparum culture specimens (samples 1 through 3) and two P. vivax-infected clinical specimens (samples 4 and 5) in 2-fold dilution (1:4, 1:8, 1:16, 1:32, 1:64, and 1:128) in sample diluent. Each dilution was treated as a test sample. Dilutional linearity was determined by accuracy, investigating the relationship between expected theoretical concentration and the observed concentration.

### Precision and accuracy.

Interassay and intra-assay precision and accuracy were determined by repeated analysis of the control samples spiked with recombinant HRP2 and P. vivax LDH or CRP at the expected concentrations (high, medium, and low), and they were evaluated by measuring each of the biomarkers in 20 tests over 2 weeks or in a single day, respectively. The precision of the method at each concentration was reported as CV%, expressing the standard deviation as a percentage of the mean calculated concentration. The accuracy was determined by expressing the observed concentration as a percentage of the expected concentration.

### Cutoff calculation for sensitivity and specificity.

Each clinical specimen was tested both neat and diluted 100-fold unless noted otherwise. To determine the optimal cutoff values for malaria antigens in pixel intensity for each biomarker, receiver operating characteristic (ROC) analysis was conducted by plotting the 1-specificity (the false-positive rate) on the *x* axis and the sensitivity (the true positive rate) on the *y* axis. At each cutoff value, sensitivity and specificity were calculated with their confidence interval. The cutoff value to yield a specificity of 98.5% was selected. Cutoff values in protein concentration were extrapolated from a long-term calibrator curve using GraphPad Prism 6.0 software (GraphPad, CA, USA).

### Data analysis.

Statistical analyses were performed to summarize data descriptively, and included means, standard deviations, and CV%. All tests for significance were two sided, and *P* values of ≤0.05 were considered significant. Statistical analyses were performed using GraphPad Prism 6.0 software.

## RESULTS

### Development of the 4-plex array.

A sensitive and quantitative multiplexed immunoassay that can simultaneously detect three Plasmodium antigens (HRP2, pan LDH, and P. vivax LDH) and CRP was developed by redesigning a previously described 2-plex array (18). Initially, several monoclonal antibody-recognizing HRP2 (including the custom-made anti-HRP2 described previously [21]), and antibodies recognizing distinct pan-species conserved and P. vivax-specific LDH epitopes were screened as capture and/or detection modalities (Table S1 in the supplemental material).

Checkerboard analysis of paired antibodies was performed to select antibody pairs with the highest signal-to-noise ratio (Tables S2 and S3 in the supplemental material). The array was optimized to maximize specificity and sensitivity and limit cross-reactivity from soluble interfering biomaterials. The final antibodies and calibrators used in the 4-plex array are shown in Table S4 in the supplemental material.

### Calibrator curves.

To determine the minimum detectable concentration and the range of quantifiable concentrations for each biomarker, the calibrator curve for each biomarker was established using the 5-parameter logistic fit. The accuracy and precision of each calibrator were examined from 12 runs. The assay results showed the acceptable range of 80% to 120% for the back-calculated concentrations of the upper 6 to 7 serial dilution points for each biomarker (Table S5 in the supplemental material). For this calibrator evaluation, LLD, LLOQ, and ULOQ values for each biomarker are shown in Table 1.

**TABLE 1:** Lower limit of detection, lower limit of quantification, and upper limit of quantification for HRP2, pan LDH, P. vivax LDH, and CRP

Target	LLD^*a*^	LLOQ^*b*^	ULOQ^*b*^
HRP2 (pg/ml)	0.2	0.9	588 (330)^*c*^
Pan LDH (pg/ml)	9.3	14.4	10,596.0
P. vivax LDH (pg/ml)	1.5	2.4	479.6
CRP (ng/ml)	26.6	44.4	9,722.8

a Lower limit of detection (LLD) = 2 × (SD of negative-control pixel intensities) × lower limit of quantification (LLOQ)/(difference between pixel intensity of lowest standard and negative control).

b LLOQ and upper limit of quantification (ULOQ) have 80% to 120% accuracy and a coefficient of variation of less than 30%.

c ULOQ established by the presence of the prozone effect. The prozone effect is defined as false-negative or false-low results due to presence of excess amounts of antigens or antibodies in immune reactions.

### Evaluation of the dilutional linearity.

Dilutional linearity of the array was examined to determine if assay results are proportional to level of dilution in assay diluent. Each of the assay results were within the acceptable range of 80% to 120% for sample dilution of up to 128-fold (Table S6 in the supplemental material).

### Evaluation of the precision and accuracy.

The results of intra-assay precision demonstrated limited variation in the mean analyte concentrations of spiked specimens, with CV% within the acceptance criterion of 10% or less, as shown in Table 2. For interassay precision, the mean analyte concentrations of spiked specimens with interassay CV% showed little variation (Table 2), with each mean CV% within the acceptable range of 0% to 20%.

**TABLE 2:** Precision and accuracy of the 4-plex array for the analysis of malaria proteins and CRP in human blood specimens

Target	Nominal concn	Intra-assay (*n* = 20)			Interassay (*n* = 20)		
		Mean Concn	Precision (CV%)^*a*^	Accuracy (%)	Mean concn	Precision (CV%)	Accuracy (%)
HRP2 (pg/ml)	23.0	22.0	6.7	95.7	21.7	11.4	94.2
	126.5	127.1	10.0	100.5	128.2	14.0	101.3
	230.0	233.0	10.3	101.3	251.3	16.0	109.3
Pan LDH (pg/ml)	450.0	379.6	5.2	84.4	407.2	8.9	90.5
	2,475.0	2,577.5	6.9	104.1	2,402.2	10.3	97.1
	4,500.0	5,120.6	5.9	113.8	4,769.9	12.9	106.0
P. vivax LDH (pg/ml)	22.8	21.0	4.2	91.9	20.9	9.0	91.6
	125.4	127.0	5.7	101.2	118.2	10.1	94.3
	228.0	246.2	5.3	108.0	229.5	11.2	100.7
CRP (ng/ml)	827.0	717.1	9.9	86.7	838.0	8.5	101.4
	3,813.0	3,590.2	8.7	94.1	3,876.9	11.6	101.7
	6,800.0	6,825.7	9.4	100.4	6,572.9	13.3	96.7

a The assay CV% for each protein concentration was calculated by finding the standard deviation of replicates, dividing that by the mean, and then multiplying by 100. The mean concentration was compared to the predetermined concentration of specimen to determine the accuracy % of the ELISA. The accuracy % was calculated as the value of (observed-expected)/expected × 100.

### Analytical evaluation of the performance.

**FIG 1:**
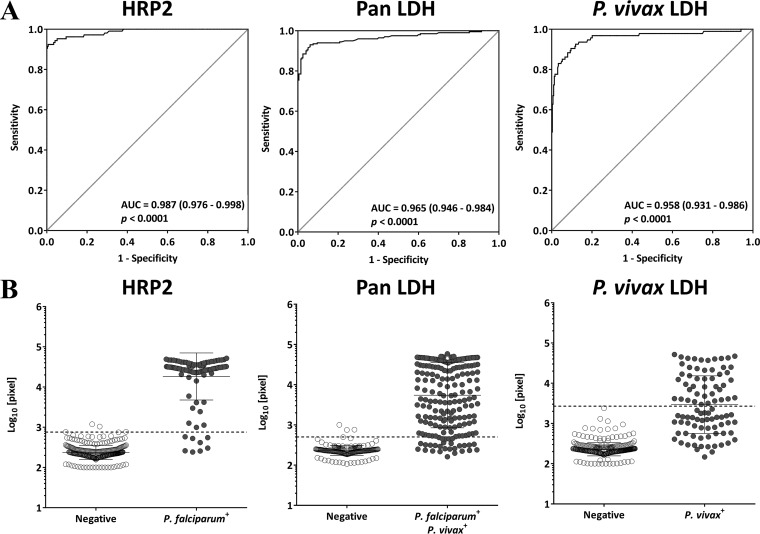
Receiver operating characteristic (ROC) curves and scatter plots depicting the reactivity of malaria antigens in Plasmodium-negative and -positive groups. The area under the concentration-time curve (AUC) of the 4-plex array for malaria antigens was conducted in reference to quantitative PCR (qPCR) methods. (A) Mean AUC plot (slanted line, black circles). The diagonal straight line represents the performance expected of a noninformative test (one with 50% sensitivity and 50% specificity). Tests were performed with blood from negative individuals (*n* = 197), P. falciparum-infected individuals (*n* = 106), and P. vivax-infected individuals (*n* = 94). (B) Reactivity of malaria infection and control blood. Scatter plots represent optical density measurement.

A total of 397 specimens were analyzed to determine the cutoff value of each assay for HRP2, pan LDH, and P. vivax LDH. To extend quantitative range for the malaria antigens, each of the clinical specimens was screened neat and at 100-fold dilution. Infection status in each specimen was determined by two different molecular tests as described previously (18, 24). The cutoff values, as defined by ROC analysis, were determined by using array pixel intensity spotted with blood specimens from P. falciparum-infected individuals for HRP2, P. falciparum- or P. vivax-infected individuals for pan LDH, and P. vivax-infected individuals for P. vivax LDH. These were compared to uninfected individuals. The area under the ROC curve (AUC) values from each ROC curve for the malarial biomarkers were 95.8% or greater (Fig. 1A). Optimal cutoffs were set for a minimum specificity of 98.5% (Fig. 1B). Cutoff values for protein concentration of 2.3 pg/ml, 47.8 pg/ml, and 75.1 pg/ml for HRP2, pan LDH, and P. vivax LDH, respectively, were used to calculate the diagnostic sensitivity and specificity of the 4-plex array, with PCR confirmation as the gold standard (Table 3).

**TABLE 3:** Performance characteristics for each malaria antigen assay

Target	Threshold	Sensitivity (95% CI)^*a*^	Specificity (95% CI)
HRP2 (pg/ml)	2.3	93.4 (86.9–97.3)	99.0 (96.4–99.9)
Pan LDH (pg/ml)	47.8	84.9 (76.7–91.1)	98.5 (95.6–99.7)
P. vivax LDH (pg/ml)	75.1	48.9 (38.5–59.5)	100 (98.8–100)
CRP (ng/ml)	NA^*b*^	NA	NA

a CI, confidence interval.

b NA, not applicable.

### Antigen dynamics in malaria infection.

**FIG 2:**
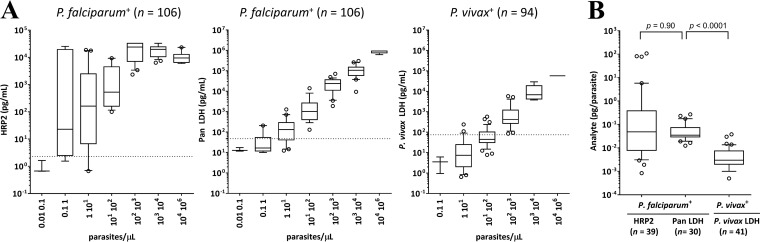
Characteristics and outcomes of the clinical field studies. (A) Distribution of HRP2 and pan LDH concentrations with respect to parasite density (0.01 to 10^6^ parasites/µl). The results show a box plot with the median (horizontal line), the 25th and 75th percentiles (boxes), the 10th and 90th percentiles (error bars), and the outliers (open circles). The dotted lines indicate cutoff values, as follows: 2.3 pg/ml for HRP2, 47.8 pg/ml for pan LDH, and 75.1 pg/ml for P. vivax LDH. The face concentration values were used in this analysis for specimens with concentration at more than ULOQ. (B) Distribution of HRP2, pan LDH, and P. vivax LDH amount per a circulating parasite respect to Plasmodium infection. Limited analysis was performed on specimens with quantifiable malaria antigens, because the accurate quantification of HRP2 is not possible at more than approximately 100 parasites/µl (P. falciparum-positive for HRP2, *n* = 39; P. falciparum-positive for pan LDH, *n* = 30; P. vivax-positive for P. vivax LDH, *n* = 41). The results show the median (horizontal line), the 25th and 75th percentiles (boxes), the 10th and 90th percentiles (error bars), and the outliers (open circles). The two-tailed Mann–Whitney U-test was used to compare the distributions of two unmatched groups.

The relationship between parasitemia and circulating malaria antigens was explored using the data set collected from asymptomatic individuals. Antigen concentrations were compared to parasite density by PCR, with confirmed single P. falciparum infections for HRP2 and P. falciparum or P. vivax infections for pan LDH. The correlation coefficient (*R*^2^) gives a crude estimation of the association between parasite density and protein concentrations. The results revealed that HRP2 showed poor correlation with parasite density (*R*^2^ = 0.45, *P* < 0.0001), whereas pan LDH showed good correlation to parasite density (*R*^2^ = 0.944, *P* < 0.0001). Importantly, a wider variation of HRP2 levels were shown in specimens within the ranges of 0.1 to 100 parasites/µl, but pan LDH and P. vivax LDH levels in P. falciparum-positive specimens and P. vivax-positive specimens showed a small variation throughout the given ranges of parasite density (Fig. 2A).

The amount of circulating HRP2 and pLDH per parasite was calculated at the low range of parasite density (0.01 to 100 parasites/µl). The median values were 0.049 pg/parasite (range, 0.001 to 108) for HRP2 in P. falciparum-positive specimens, 0.035 pg/parasite (range, 0.013 to 0.269) for pan LDH in P. falciparum-positive specimens, and 0.003 pg/parasite (range, 0.001 to 0.038) for P. vivax LDH in P. vivax-positive specimens (Fig. 2B, Table S7 in the supplemental material). In this specimen set, there was no statistical difference between the amounts of HRP2 and pan LDH antigens per parasite for P. falciparum (*P* = 0.9). The amount of P. vivax LDH per circulating parasite was statistically significantly lower for P. vivax parasites than the pan LDH for P. falciparum parasites (*P* < 0.0001).

### Antigen dynamics in induced blood-stage malaria infection.

**FIG 3:**
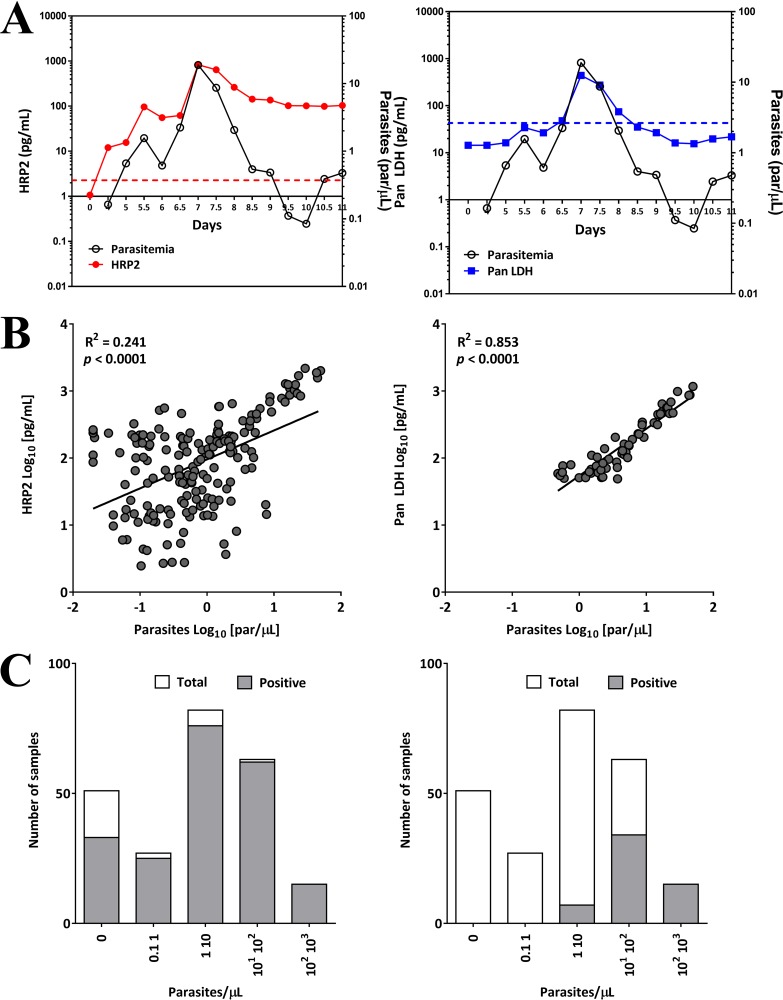
Characteristics and outcomes of the IBSM study. (A) Time course of parasitemia and malaria antigens from days 0 through 11 in volunteers inoculated with P. falciparum parasites. Antigen concentration and parasitemia were plotted as the means of measurements of specimens from 16 individuals for each time point. The dotted lines indicate cutoff values, namely, 2.3 pg/ml for HRP2 and 47.8 pg/ml for pan LDH. (B) Relationship between parasitemia and concentration of malaria proteins from specimens. Samples that were positive by qPCR, and the 4-plex array for HRP2 (left), and pan LDH (right) are presented. The *R*^2^ values were found to be 0.241 and 0.853 for HRP2 (*n* = 178) and pan LDH (*n* = 56), respectively. (C) Distributions of parasitemia levels for antigen-positive specimens out of 238 samples for HRP2 (left) and pan LDH (right).

To assess parasite clearance and detection of malaria antigens after antimalaria treatment, induced blood-stage malaria (IBSM) infections were investigated. IBSM allows malaria infection to be done in a controlled environment (18). Sixteen malaria-naive volunteers were intravenously inoculated with P. falciparum 3D7 parasites on day 0 and treated on day 7 with an antimalarial drug. From days 0 through 11, 238 specimens from these volunteers were tested by both qPCR and the 4-plex array. The mean concentration for each malaria antigen was compared to the mean parasitemia as measured by qPCR. Both HRP2 and pan LDH antigens had similar kinetics, with an increase in concentration toward day 7 and a subsequent decrease posttreatment (Fig. 3A). However, pan LDH concentration decreased more rapidly after day 8, while HRP2 declined slowly and was still detectable on day 11 in 6 out of 7 (85.7%) subjects with cleared parasitemia (Fig. S1A in the supplemental material). The relationships between concentration of each of the malaria antigens and the parasite density were examined in specimens collected on days 4 through 11. Good correlation was seen for pan LDH (*R*^2^ = 0.853), while HRP2 showed poor correlation (*R*^2^ = 0.241) (Fig. 3B). The impact of persistent HRP2 presence, even after parasite clearance, was examined next. The distributions of the specimens sorted by parasite density are shown in Fig. 3C. Of the total (238), 187 specimens confirmed parasite positive by qPCR, 178 (95.2%) were positive for HRP2, and 56 (29.9%) were positive for pan LDH by the 4-plex array. None of the samples that were qPCR negative tested antigen positive for pan LDH. In contrast, 31 qPCR-confirmed parasite cleared samples collected after clearance tested as HRP2 antigen positive (Fig. S1B in the supplemental material).

### Association between CRP and infection.

CRP levels of noninfected individuals were compared to levels in infected individuals to assess the relationship between CRP levels and low parasite density malaria infection. There was no significant difference in the CRP levels of the two groups as determined by qPCR (*P* = 0.053) (Fig. S2A in the supplemental material). However, median values of CRP from the HRP2-positive subgroup (*P* < 0.01) and pan LDH-positive subgroup (*P* < 0.005) of P. falciparum specimens were statistically significantly higher than that of the noninfected group (Fig. S2B in the supplemental material). The same was not observed for P. vivax.

## DISCUSSION

A new diagnostic tool that can quantify the two most commonly used antigens in malaria RDTs, HRP2 and pLDH, as well as the inflammation marker CRP, from the same blood sample was developed. While assays for pLDH or highly sensitive assays for HRP2 have been described previously (16, 26, 27), the 4-plex array is capable of measuring both HRP2 and pLDH antigens at low concentrations and discriminating between two Plasmodium species with no cross-reactivity (Table 2). The 4-plex array is comparable in LOD to these highly sensitive bead-based assays for HRP2, which range in LOD from 0.24 pg/ml to 70 pg/ml, depending on the HRP2 type detected (16). In the case of RDTs, the ultrasensitive HRP2-based RDT has an LOD of 40 pg/ml to 80 pg/ml (18, 19), while the best conventional RDTs have LODs for HRP2 and pLDH on the order of 800 pg/ml and 2.9 ng/ml, respectively (28). As RDTs improve in LOD for malaria antigens it is important to be able to confirm true positives or negatives for antigenemia with an independent assay with a lower LOD than the RDT, such as the 4-plex array. The utility of the 4-plex array for the evaluation of both current and new, ultrasensitive RDTs for use in malaria control and elimination has already been demonstrated in an evaluation of the new ultrasensitive HRP2-based RDT in Myanmar (29).

Two significant challenges encountered in the development of the quantitative assays for pLDH and HRP2 were the observation of the prozone effect in the HRP2 assay and the wide variability in the reactivity of antibodies to different sources of pLDH. First, the prozone effect resulting in dampening signal at high HRP2 concentrations that caused narrowing of the dynamic range of this assay. This phenomenon has also been observed in HRP2-based RDTs (30–32). This limitation was addressed with sample dilution. Second, extreme variability in the pLDH assay performance depending on the recombinant pLDH antigen source, likely due to differences in protein folding efficiency and/or epitope sequences in pLDH, resulted in challenges in the assay calibration and performance evaluation. As a result, since the pan LDH signal was calibrated against P. falciparum standards, the accuracy of the quantification for pan LDH is likely to be lower for other species and needs further investigation. Typical factors that can interfere with malaria immunoassays, such as lipids, bilirubin, and hematocrit, did not interfere with the assay performance (data not shown). The effects of other potentially interfering factors, such as rheumatoid factor, hepatitis C, toxoplasmosis, human African trypanosomiasis, dengue, leishmaniasis, Chagas disease, and schistosomiasis have yet to be investigated.

Good positive correlation of pLDH concentration and parasite density was observed in specimens collected from Uganda and Myanmar, but the HRP2 concentration correlated poorly with parasitemia for the same specimens. These results were confirmed in the IBSM study (Fig. 3). Good correlation between pLDH concentration and parasitemia has been observed previously (33). This difference in relationship of antigen levels to parasitemia is likely the result of the different half-lives of the two antigens in the blood. pLDH is quickly cleared from the bloodstream after cure of malaria, whereas HRP2 persists for some time (34–36). There are ongoing efforts to model the dynamics of the HRP2 and pLDH based on results from this assay and IBSM studies.

The HRP2 assay achieved a lower LOD than that of the pLDH assay, possibly as a result of the availability of antibodies with better binding kinetics and larger numbers of epitopes per molecule. Despite this, within the constraints of the number of clinical specimens tested for this study, it was observed that HRP2 and pan LDH levels in P. falciparum were equivalent. However, P. vivax LDH levels were statistically lower in P. vivax compared to pan LDH levels in P. falciparum samples when normalized for parasite count. The poor sensitivity observed in RDTs for P. vivax malaria is likely the compound result of poor assay reagents, low parasite densities in infection, and relatively low availability of antigen per parasite. In future studies, the root cause may be resolved through quantitative proteomic analysis via matrix-assisted laser desorption ionization–time of flight mass spectrometry (MALDI-TOF MS). Development of more sensitive tests for P. vivax malaria will need to take these considerations into account.

CRP is often used as an inflammatory biomarker that is indicative of malaria parasitemia and other infections associated with febrile illness (14, 15). In this study, CRP levels were moderately elevated only in asymptomatic P. falciparum infections with confirmed antigenemia (either HRP2 or pan LDH). The increase was not statistically significant in confirmed parasite infections for the same sample set. Likewise, no significant differences were observed in CRP levels for P. vivax infection by parasitemia or antigenemia. Further investigation with a larger sample set is necessary to understand the association between CRP levels and low parasite density infections.

The 4-plex array is a useful tool for further characterizing low density infections, which prevail in settings approaching elimination and have significant transmission potential. The tool is also essential to validate the performance of new ultrasensitive RDTs and to inform their impact in malaria elimination strategies (16, 18, 19, 37). This multiplex array is currently being expanded to include additional parasite and host biomarkers to inform the relationship between low-density infection and disease, as well as the development of the next generation of diagnostics to support malaria elimination.

## Supplementary Material

Supplemental file 1
