# Supplementary material for: Simultaneous Quantification of Plasmodium Antigens and Host Factor C-Reactive Protein in Asymptomatic Individuals with Confirmed Malaria by Use of a Novel Multiplex Immunoassay
Source: J Clin Microbiol. 2019 Jan 2;57(1):e00948-18. doi: 10.1128/JCM.00948-18 (PMC6322473; doi:10.1128/JCM.00948-18)
Supplement: Supplemental file 1 [file e6a64e6f07e50b64fd386546d6aec09f_JCM.00948-18-s0001.pdf]

SUPPLEMENTAL MATERIAL

Table of contents:

Table S1. *Plasmodium* antigen-specific monoclonal antibodies used in screening for the 4-Plex array development………………………………………………………………………………………… S-2

Table S2. Rapid antibody pairing to identify the high performing antibody pair in detecting HRP2.... S-3

Table S3. Rapid antibody pairing to identify the high performing antibody pair in detecting Pan LDH and *P. vivax* LDH…………………………………………………………………………...………. S-4

Table S4. Final monoclonal antibodies and calibrator proteins with their sources used on the 4-Plex array ……………………………………………………………………………………………...S-5

Table S5. Characterization of calibrator curves generated during assay validation…………………… S-6

Table S6. Dilutional linearity with the 4-Plex array……………………………………..………...... S-7

Table S7. Descriptive statistics for amount of circulating antigen per parasite…………….…….… S-9

Fig S1. Parasitemia and concentration of HRP2 and Pan LDH in blood specimens from all subjects obtained during a course of IBSM study…………………………..………………………………. S-10

Fig S2. Distribution of CRP levels in malaria non-infected and infected groups…….……...…… S-11

Table S1. *Plasmodium* antigen-specific monoclonal antibodies used in screening for the 4-Plex array development

Biomarker	Clone	Host	Property	Ig class	Supplier	Applications
HRP2	MPFM-55A	Mouse	Monoclonal	IgM	Immunology consultants lab	ELISA, LF, WB
	MPFG-55A	Mouse	Monoclonal	IgG	Immunology consultants lab	ELISA, LF, WB
	PTL3	Mouse	Monoclonal	IgM	National Bioproducts Institute	ELISA, LF, WB
	C1-13	Mouse	Monoclonal	IgG	National Bioproducts Institute	ELISA, LF, WB
	0445	Mouse	Monoclonal	IgG	Vista Diagnostics	ELISA
	2G6	Mouse	Monoclonal	IgG	Vista Diagnostics	ELISA
	4D6	Mouse	Monoclonal	IgG	Precision Antibody	ELISA
	6C8	Mouse	Monoclonal	IgG	Precision Antibody	ELISA
	8D3	Mouse	Monoclonal	IgG	Precision Antibody	ELISA
	10C1	Mouse	Monoclonal	IgG	Precision Antibody	ELISA
	10F5	Mouse	Monoclonal	IgG	Precision Antibody	ELISA
	11E10	Mouse	Monoclonal	IgG	Precision Antibody	ELISA
	12D4	Mouse	Monoclonal	IgG	Precision Antibody	ELISA
	12F12	Mouse	Monoclonal	IgG	Precision Antibody	ELISA
Pan LDH	1201	Mouse	Monoclonal	IgG	Vista Diagnostics	ELISA
	19G7	Mouse	Monoclonal	IgG	Vista Diagnostics	ELISA
	1246	Mouse	Monoclonal	IgG	Vista Diagnostics	ELISA
	12G1	Mouse	Monoclonal	IgG	Vista Diagnostics	ELISA
	6G9	Mouse	Monoclonal	IgG	Vista Diagnostics	ELISA
P. vivax LDH	3H8	Mouse	Monoclonal	IgG	Vista Diagnostics	ELISA
	1102	Mouse	Monoclonal	IgG	Vista Diagnostics	ELISA
	13H4	Mouse	Monoclonal	IgG	Vista Diagnostics	ELISA

Abbreviation: ELISA, enzyme-linked immunosorbent assay; LF, lateral flow; WB, western blot

Table S2. Rapid antibody pairing to identify the high performing antibody pair in detecting HRP2

	Signal-to-noise ratio of 10 pg/mL to blank; HRP2													
Capture \ Detection	MPFM-55A	MPFG-55A	PTL3	C1-13	0445	2G6	4D6	6C8	8D3	10C1	10F5	11E10	12D4	12F12
MPFM-55A	1.4	15.3	1.3	6.3	3.1	5.1	2.5	16.9	4.2	4.9	25.6	40.4	26.5	5.6
MPFG-55A	4.3	5.5	1.9	5.8	2.8	8.5	1.4	8.3	1.5	4.6	16.5	22.4	8.7	4.7
PTL3	1.7	10.8	1.2	5.9	3.6	3.7	2.6	12.4	2.0	2.4	21.3	24.9	16.2	8.4
C1-13	3.9	7.2	1.5	4.9	2.3	4.5	2.0	5.1	1.1	2.9	13.2	14.0	5.7	5.6
445	5.0	5.7	1.4	5.4	3.1	14.5	3.1	9.5	1.3	4.0	20.2	31.5	13.3	5.9
2G6	2.4	12.7	1.0	2.9	3.0	8.2	1.5	18.7	4.0	2.1	41.7	55.3	10.4	8.2
4D6	2.5	2.5	1.0	3.2	2.0	8.0	1.0	6.0	2.1	1.0	9.9	20.6	1.2	1.9
6C8	1.0	1.3	0.9	1.1	1.3	3.4	0.9	1.5	1.2	1.0	1.5	4.2	1.0	0.9
8D3	3.9	6.0	1.3	5.0	2.4	14.2	2.2	7.2	1.1	2.5	18.3	25.3	3.7	4.4
10C1	3.5	3.9	0.9	4.0	2.0	8.5	1.5	6.4	3.9	2.4	14.8	33.3	4.4	4.5
10F5	2.5	2.3	0.9	3.3	2.0	7.3	2.4	7.5	2.6	2.8	15.2	25.1	8.1	5.4
11E10	1.2	1.5	1.0	1.1	1.5	2.7	1.0	2.3	2.4	1.0	1.2	2.5	1.0	1.0
12D4	3.3	3.4	1.4	4.4	2.3	8.9	1.2	7.6	2.4	1.8	19.2	26.0	1.7	3.0
12F12	3.0	3.1	1.1	4.0	1.9	6.7	1.5	9.5	2.5	2.1	17.2	32.8	3.2	5.2

For the rapid antibody pairing study, an array immobilized with 14 of anti-HRP2 monoclonal antibodies on the bottom of each well of 96-well plate was screened pairwise against 10, 100, and 1,000 pg/ml of recombinant HRP2 protein. The performance of each capture-detection pair was screened by measuring signal-to-noise ratio. Signal-to-noise ratio of 10 pg/ml to blank sample is shown. The heat map was created using the Quick Analysis tool from Excel. The red in color represents the stronger binding response whereas the blue for the poor binding response.

Table S3. Rapid antibody pairing to identify the high performing antibody pair in detecting Pan LDH and *P. vivax* LDH

	Signal-to-noise ratio of 1000 pg/ml to blank; *P. falciparum* LDH							
Capture \ Detection	1201	19G7	1246	12G1	6G9	3H8	1102	13H1
1201	57.4	27.6	62.8	16.0	9.6	0.9	1.0	0.9
19G7	133.3	2.4	39.9	4.0	16.8	1.0	1.1	1.0
1246	38.1	12.6	47.5	1.5	8.4	0.9	1.0	1.0
12G1	103.6	4.2	59.1	1.7	23.5	1.0	0.8	0.9
6G9	16.7	6.3	19.9	3.7	3.1	1.0	0.9	1.0
3H8	1.1	0.9	1.5	1.0	1.1	1.2	1.0	1.1
1102	1.4	0.9	0.9	0.9	0.8	0.9	1.2	1.0
13H1	1.0	1.2	1.3	0.9	0.8	1.1	0.8	1.2
	Signal-to-noise ratio of 1000 pg/ml to blank; *P. vivax* LDH							
Capture \ Detection	1201	19G7	1246	12G1	6G9	3H8	1102	13H1
1201	46.0	34.0	10.1	24.1	1.4	39.9	56.2	9.0
19G7	113.1	4.0	8.8	12.6	1.7	37.7	21.7	14.1
1246	42.1	14.7	6.0	2.2	1.3	17.1	6.2	3.8
12G1	169.3	5.5	13.5	2.0	1.6	30.5	1.0	2.0
6G9	9.8	4.7	2.2	4.2	1.1	6.3	1.0	1.9
3H8	41.6	4.7	3.3	2.1	1.0	2.1	1.0	1.0
1102	52.7	5.5	4.0	1.2	0.9	1.1	4.2	1.3
13H1	37.5	13.0	2.2	6.9	1.0	1.8	1.3	2.0

For the rapid antibody pairing study, an array immobilized with 8 of anti-pan LDH and anti-*P. vivax* LDH monoclonal antibodies on the bottom of each well of 96-well plate was screened pairwise against 100, 1,000, and 10,000 pg/ml of either *P. falciparum* LDH or *P. vivax* LDH proteins. The performance of each capture-detection pair was screened by measuring signal-to-noise ratio. Signal-to-noise ratio of 1,000 pg/ml to blank sample is shown. The heat map was created using the Quick Analysis tool from Excel. The red in color represents the stronger binding response whereas the blue for the poor binding response.

Table S4. Final monoclonal antibodies and calibrator proteins with their sources used on the 4-Plex array

Biomarker	Capture antibody	Detection antibody	Calibrato	Assay
HRP2	0445 (Vista)	11E10 (Precision)	HRP2 (Microcoat)	Quantitative
Pan LDH	12G1 (Vista)	1201 (Vista)	*P. vivax* LDH (CTK)	Qualitative[b]/Quantitative
P. vivax LDH	1102 (Vista)	1201 (Vista)	*P. vivax* LDH (CTK)	Quantitative
Human CRP	C6 (HyTest)	n.a [a]	CRP (HyTest)	Quantitative

[a] Quantification of human CRP is based on competitive ELISA using biotinylated CRP. Supplier is shown in parenthesis.

[b] Pan LDH assay may be used for quantification of pLDH in a specimen infected with *P. falciparum* species.

Table S5. Characterization of calibrator curves generated during assay validation

HRP2 (pg/mL)	Pixel intensity Mean, CV%		Back-calculated Mean, CV%		Accuracy %[a]	Pan LDH (pg/mL)	Pixel intensity Mean, CV%		Back-calculated Mean, CV%		Accuracy %[a]
590	47833.3	1.7	588.0	28.5	99.7	10,514	33431.5	5.7	10596.0	11.2	100.8
196.7	41176.1	2.5	190.8	8.0	97.0	3,504.7	15460.0	9.3	3480.3	10.5	99.3
65.6	22433.3	6.9	66.8	8.2	101.8	1,168.2	5571.2	8.2	1155.5	9.0	98.9
21.9	7747.8	8.5	21.6	8.5	98.8	389.4	2023.5	7.9	390.8	9.1	100.4
7.3	2692.3	9.7	7.5	9.9	102.3	129.8	798.9	8.8	128.7	11.3	99.1
2.4	907.7	16.9	2.3	20.2	93.7	43.3	388.8	7.8	42.2	16.0	97.5
0.8	450.2	14.9	0.9	23.8	109.0	14.4	259.5	6.9	14.4	26.8	100.1
0.3	252.7	25.0	0.3	72.4	101.4	4.8	213.2	13.9	7.3	65.8	151.7

P. vivax LDH (pg/mL)	Pixel intensity Mean, CV%		Back-calculated Mean, CV%		Accuracy %[a]	CRP (ng/mL)	Pixel intensity Mean, CV%		Back-calculated Mean, CV%		Accuracy %[a]
497.0	10365.1	8.2	479.6	9.9	96.5	13,500	496.7	9.4	13713.3	11.2	101.6
165.7	3990.5	9.1	165.5	10.0	99.9	4,500	1278.0	12.3	4479.7	16.8	99.5
55.2	1457.0	8.1	54.7	9.9	99.0	1,500	3109.5	11.0	1543.4	13.8	102.9
18.4	637.1	6.2	18.7	9.7	101.4	500	7732.9	10.2	505.4	13.1	101.1
6.1	347.8	8.0	6.1	20.6	99.1	166.7	16965.8	10.2	159.8	18.8	95.9
2.1	251.0	10.2	2.4	29.6	115.1	55.6	24216.3	6.2	61.5	28.7	110.6
0.7	223.0	14.2	1.3	81.3	193.5	18.5	27828.5	9.0	37.3	73.6	201.2
0.2	209.7	15.8	1.9	26.9	819.4	6.2	28755.7	9.2	35.9	57.6	581.8

[a] Accuracy was calculated using the formula; observed concentration/expected concentration) x 100. Data were analyzed from twelve calibration curves obtained on different days.

Table S6. Dilutional linearity with the 4-Plex array

		HRP2			Pan LDH		
		Expected conc. (pg/mL)	Mean adjusted conc. (pg/mL)	Accuracy %	Expected conc. (pg/mL)	Mean adjusted conc. (pg/mL)	Accuracy %
Sample 1	1:4	45.0	38.2	84.9	55.0	53.2	96.8
	1:8	22.5	22.1	98.2	27.5	27.8	101.1
	1:16	11.3	11.2	99.2	13.8	<LLOQ	
	1:32	5.6	5.8	102.6	6.9	<LLOQ	
	1:64	2.8	3.0	106.5	3.4	<LLOQ	
	1:128	1.4	1.6	112.0	1.7	<LLOQ	
Sample 2	1:4	325.0	332.3	102.2	300.0	267.0	89.0
	1:8	162.5	167.2	102.9	150.0	140.1	93.4
	1:16	81.3	80.1	98.5	75.0	72.1	96.2
	1:32	40.6	39.5	97.1	37.5	37.9	101.1
	1:64	20.3	19.5	95.9	18.8	18.9	100.7
	1:128	10.2	10.1	99.5	9.4	<LLOQ	
Sample 3	1:4	275.0	318.7	115.9	200.0	174.4	87.2
	1:8	137.5	135.5	98.5	100.0	95.9	95.9
	1:16	68.8	65.9	95.9	50.0	51.8	103.6
	1:32	34.4	32.1	93.3	25.0	25.0	99.8
	1:64	17.2	16.3	94.8	12.5	<LLOQ	
	1:128	8.6	8.6	100.2	6.3	<LLOQ	
Sample 4	1:4	ND	<LLOQ		875.0	831.5	95.0
	1:8	ND	<LLOQ		437.5	446.0	101.9
	1:16	ND	<LLOQ		218.8	215.5	98.5
	1:32	ND	<LLOQ		109.4	110.0	100.6
	1:64	ND	<LLOQ		54.7	55.6	101.6
	1:128	ND	<LLOQ		27.3	29.5	107.8
Sample 5	1:4	ND	<LLOQ		1040.7	1050.0	99.1
	1:8	ND	<LLOQ		532.0	525.0	101.3
	1:16	ND	<LLOQ		253.3	262.5	96.5
	1:32	ND	<LLOQ		129.1	131.3	98.4
	1:64	ND	<LLOQ		62.2	65.6	94.8
	1:128	ND	<LLOQ		38.0	32.8	115.9

		P. vivax LDH			CRP		
		Expected conc. (pg/mL)	Mean adjusted conc. (pg/mL)	Accuracy %	Expected conc. (pg/mL)	Mean adjusted conc. (pg/mL)	Accuracy %
Sample 1	1:4	ND	<LLOQ		ND	<LLOQ	
	1:8	ND	<LLOQ		ND	<LLOQ	
	1:16	ND	<LLOQ		ND	<LLOQ	
	1:32	ND	<LLOQ		ND	<LLOQ	
	1:64	ND	<LLOQ		ND	<LLOQ	
	1:128	ND	<LLOQ		ND	<LLOQ	
Sample 2	1:4	ND	<LLOQ		70.0	69.5	99.2
	1:8	ND	<LLOQ		35.0	<LLOQ	
	1:16	ND	<LLOQ		17.5	<LLOQ	
	1:32	ND	<LLOQ		8.8	<LLOQ	
	1:64	ND	<LLOQ		4.4	<LLOQ	
	1:128	ND	<LLOQ		2.2	<LLOQ	
Sample 3	1:4	ND	<LLOQ		450.0	495.3	110.1
	1:8	ND	<LLOQ		225.0	200.7	89.2
	1:16	ND	<LLOQ		112.5	<LLOQ	
	1:32	ND	<LLOQ		56.3	<LLOQ	
	1:64	ND	<LLOQ		28.1	<LLOQ	
	1:128	ND	<LLOQ		14.1	<LLOQ	
Sample 4	1:4	223.3	230.6	103.3	100.0	103.6	103.6
	1:8	111.6	120.1	107.6	50.0	<LLOQ	
	1:16	55.8	54.4	97.5	25.0	<LLOQ	
	1:32	27.9	28.1	100.6	12.5	<LLOQ	
	1:64	13.9	13.2	94.8	6.3	<LLOQ	
	1:128	7.0	7.1	102.5	3.1	<LLOQ	
Sample 5	1:4	266.0	296.4	111.4	ND	<LLOQ	
	1:8	133.0	145.2	109.2	ND	<LLOQ	
	1:16	66.5	65.2	98.0	ND	<LLOQ	
	1:32	33.3	31.4	94.4	ND	<LLOQ	
	1:64	16.6	14.8	89.0	ND	<LLOQ	
	1:128	8.3	8.1	97.9	ND	<LLOQ	

[a] Accuracy was calculated using the analyte concentration determined at dilution 1:4 as a reference value.

ND: not detected

Table S7. Descriptive statistics for amount of circulating antigen per parasite

Biomarker	No. of specimen[a]	Mean	Min	25 percentile	Median	75 percentile	Max
HRP2	*39*	7.295	0.001	0.008	0.049	0.386	108
Pan LDH	30	0.066	0.013	0.030	0.035	0.074	0.269
P. vivax LDH	41	0.006	0.001	0.002	0.003	0.008	0.038

[a] Analysis was performed with specimens with parasitemia in the range of 1 to 100 parasites/μL and analyte concentration greater than the assay cutoff.

a

A B

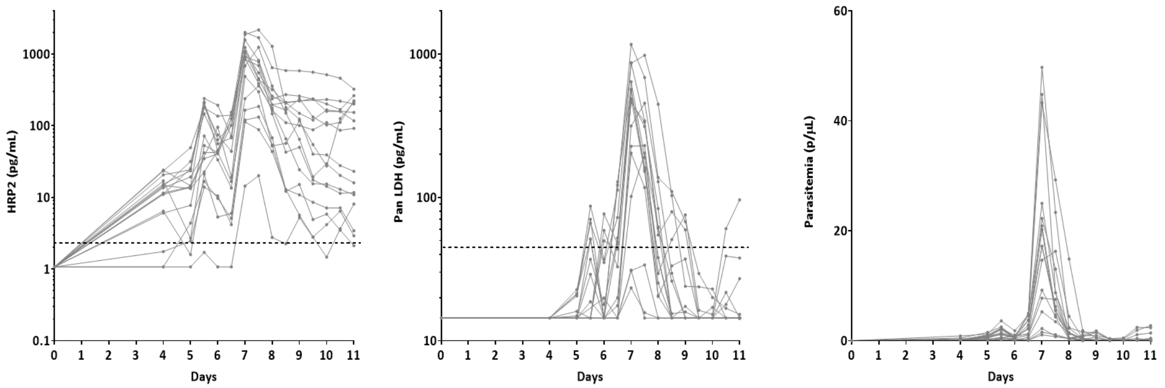

Fig S1. Concentration of HRP2 and Pan LDH, and parasitemia in blood specimens from all subjects obtained during a course of IBSM study. (A) concentration of HRP2 and Pan LDH, and (B) Parasitemia. The dotted lines indicate cut-off values.

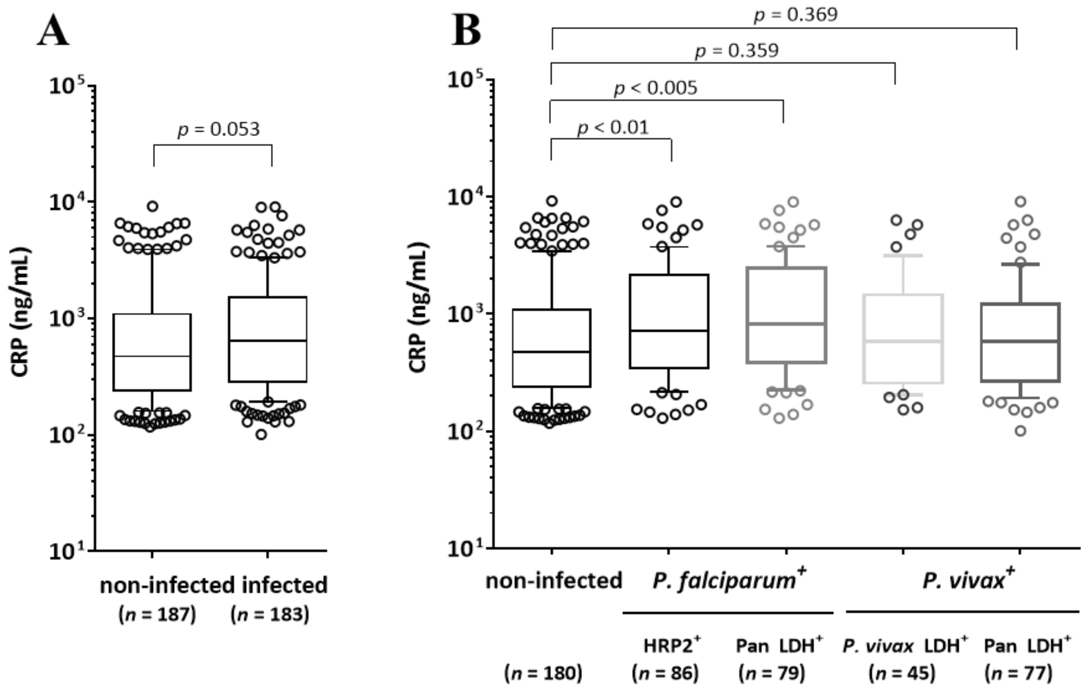

Fig S2. Distribution of CRP levels in malaria non-infected and infected groups. Box plots of CRP levels in infection positive and negative groups determined by A) qPCR and B) 4-Plex array. Uninfected group determined by 4-Plex array was defined by the absence of any of malaria antigens. CRP levels were analyzed only from specimens which had quantifiable amount of HRP2, and *P. vivax* LDH greater than the assay cutoff. The results show the median (horizontal line), the 25th and 75the percentiles (boxes), the 10th and 90th percentiles (error bars) and the outliers (open circles). The two-tailed Mann–Whitney U-test was used to compare the distributions of two unmatched groups.
